# Contribution of 3D genome topological domains to genetic risk of cancers: a genome-wide computational study

**DOI:** 10.1186/s40246-022-00375-2

**Published:** 2022-01-11

**Authors:** Kim Philipp Jablonski, Leopold Carron, Julien Mozziconacci, Thierry Forné, Marc-Thorsten Hütt, Annick Lesne

**Affiliations:** 1 Department of Biosystems Science and Engineering, ETH Zurich, 4058 Basel, Switzerland; 2 SIB Swiss Institute of Bioinformatics, 4058 Basel, Switzerland; 3 Laboratoire de Physique Théorique de la Matière Condensée, LPTMC, CNRS, Sorbonne Université, Paris, France; 4 Present Address: Laboratory of Computational and Quantitative Biology, LCQB, Sorbonne Université, Paris, France; 5 Structure et Instabilité des Génomes, Muséum National d’Histoire Naturelle, Paris, France; 6 Institut de Génétique Moléculaire de Montpellier, IGMM, CNRS, Univ. Montpellier, Montpellier, France; 7 Department of Life Sciences and Chemistry, Jacobs University Bremen, Bremen, Germany

## Abstract

**Background:**

Genome-wide association studies have identified statistical associations between various diseases, including cancers, and a large number of single-nucleotide polymorphisms (SNPs). However, they provide no direct explanation of the mechanisms underlying the association. Based on the recent discovery that changes in three-dimensional genome organization may have functional consequences on gene regulation favoring diseases, we investigated systematically the genome-wide distribution of disease-associated SNPs with respect to a specific feature of 3D genome organization: topologically associating domains (TADs) and their borders.

**Results:**

For each of 449 diseases, we tested whether the associated SNPs are present in TAD borders more often than observed by chance, where chance (i.e., the null model in statistical terms) corresponds to the same number of pointwise loci drawn at random either in the entire genome, or in the entire set of disease-associated SNPs listed in the GWAS catalog. Our analysis shows that a fraction of diseases displays such a preferential localization of their risk loci. Moreover, cancers are relatively more frequent among these diseases, and this predominance is generally enhanced when considering only intergenic SNPs. The structure of SNP-based diseasome networks confirms that localization of risk loci in TAD borders differs between cancers and non-cancer diseases. Furthermore, different TAD border enrichments are observed in embryonic stem cells and differentiated cells, consistent with changes in topological domains along embryogenesis and delineating their contribution to disease risk.

**Conclusions:**

Our results suggest that, for certain diseases, part of the genetic risk lies in a local genetic variation affecting the genome partitioning in topologically insulated domains. Investigating this possible contribution to genetic risk is particularly relevant in cancers. This study thus opens a way of interpreting genome-wide association studies, by distinguishing two types of disease-associated SNPs: one with an effect on an individual gene, the other acting in interplay with 3D genome organization.

**Supplementary Information:**

The online version contains supplementary material available at 10.1186/s40246-022-00375-2.

## Background

Genome-wide association studies (GWAS) have compared the genomes of large cohorts of patients and healthy individuals and evidenced statistical associations between the presence of single-nucleotide polymorphisms (SNPs) in their variant form, and the presence of diseases [1, 2], including cancers [3, 4]. Remarkably, only a few percent of disease-associated SNPs (daSNPs) are located in coding regions of the genome [5, 6]. How the vast majority of non-coding daSNPs are mechanistically related to the risk of developing a disease is yet unclear. While SNPs were at first considered as mere markers of the nearest gene, it rapidly appeared that they can have a direct functional role in affecting the regulation of neighboring genes, typically by being located in regulatory sequences [6, 7]. Systematic analyses of SNP location with respect to genome annotations such as binding sites of regulatory proteins or histone epigenetic marks correlated with promoter or enhancer loci [8, 9], as well as joint transcriptome analysis [10], have been successfully used to identify causal variants and affected genes.

However, such correlation analyses are not sufficient to unravel all the determinants of SNP-disease associations. An additional ingredient, the three-dimensional (3D) genome organization, must be taken into account. Indeed, it is now acknowledged that not only the adjacent sequences of the gene, but also the 3D folding of the genome play a role in genomic functions [11, 12], and more specifically in the regulation of gene expression [13] and its mis-regulation, e.g., in cancers [14]. It thus appears essential to reformulate the notion of genetic risk to a disease in the context of the recently acquired knowledge about 3D genome organization. Our working hypothesis is that certain non-coding SNPs could, in their variant form, affect the 3D genome architecture and its role in gene regulation, thus favoring the development of diseases.

This idea has been documented experimentally for enhancer-promoter loops [15]. A variant at a single SNP may induce a change in the looping bringing enhancer and promoter into close spatial proximity, henceforth affecting the expression of the corresponding genes. For instance, a single SNP may modify a CTCF binding site and in turn nucleosome positioning and chromatin 3D architecture, a documented situation for asthma risk [16, 17]. At the *MYB* locus, 3C experiments have shown reduced interactions between promoter and enhancer in the presence of the at-risk allele, providing an instance of a SNP having a causal architectural effect [18].

**Fig. 1:**
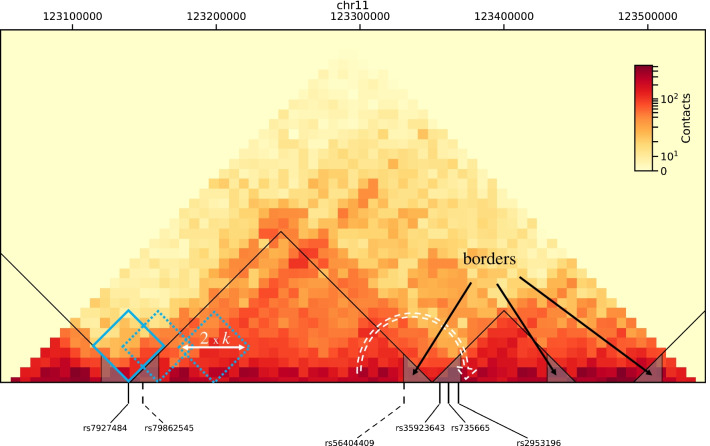
Data structure behind the investigation. Underlying Hi-C data are displayed as a heat map (the redder the more contacts, as indicated in the color bar), here for a region of chromosome 11 (chr11: 123,050,000–123,550,000, hg19 coordinates), drawn from data published in [30], for IMR90 cell type, at 10 kb-resolution. TADs are underlined with black triangle lines. They are determined using TopDom algorithm [31], which identifies a demarcation between two TADs as a local minimum of the number of contacts in a sliding window of half-size *k* bins, where *k* is a tunable parameter (blue diamonds, the full-lined one corresponding to the limit of a TAD). TAD borders are defined as 20 kb-regions from TAD ends inward, and underlined here as small triangles filled in gray. Vertical lines pinpoint disease-associated SNPs located in TAD borders (full line for cancer-associated SNPs, dashed lines for SNPs associated with non-cancer diseases). For each cancer or each non-cancer disease, we investigate the potential over-representation of its associated SNPs in TAD borders. The dashed white arrow indicates increased physical contacts and increased regulatory interactions between adjacent TADs that would possibly appear in a border affected by the presence of an at-risk SNP allele

Pursuing this line of investigation at the genome-wide level, we will consider another feature of 3D genome structure, namely topologically associating domains, TADs [19]. There are few genomic contacts between two adjacent TADs, and the insulating capacity of TAD borders [20] has been shown to be essential for proper gene regulation, by preventing spurious interactions between genes and enhancers located in adjacent TADs [21–23]. Gene mis-regulation can occur due to TAD border disruption induced by the presence of short tandem repeats [24]. The importance of TADs and the effect of TAD border disruption (dashed arrow in Fig. 1) have also been shown in the case of *Hox* genes [25], or as a way to control developmental genes in drosophila embryos, as we recently proposed [26]. An effect of TAD border disruption has also been observed in cancer cells as a consequence of cancerous mutations [27, 28].

The guideline of our computational study is that functional mechanisms underlying the association of risk loci with a disease may, in some cases, be mediated by a change occurring in TAD borders when embedded SNPs are in their at-risk-form. Accordingly, the increased disease risk due to the presence of non-coding SNPs in their variant form could presumably be better understood by investigating their location with respect to various features of the 3D genome organization, in particular its partitioning into TADs [23].

We studied quantitatively and systematically, for each disease, the location of disease-associated SNPs with respect to TAD borders (Fig. 1). Different cell types and data obtained in different laboratories have been considered (overall 15 datasets). Based on a preliminary analysis [29] and on their different etiology, we analyzed separately 71 cancers and 378 non-cancer diseases and compared the distribution of their associated SNPs with respect to TAD borders. We analyzed, for each disease, the distribution of its disease-associated SNPs in TAD borders compared to chance, where chance corresponds to the same number of pointwise loci drawn at random either in the entire genome or in the set of SNPs listed in the GWAS catalog. We investigated whether the results persist when considering only intergenic SNPs (40% of the total set of daSNPs, overall comprising 21,183 entries). An integrated pipeline, sketched in Additional file 1: Fig. S1, has been devised for this analysis, and its different elements are detailed in the Methods section.

We emphasize that the present investigation does not consider somatic mutations appearing in cancer cells along cancer progression. It focuses on genomic variations observed from birth in the genome of any healthy cell of the individual.

## Results

### For a fraction of diseases, the associated SNPs are preferentially located in TAD borders

We used 15 high-resolution published Hi-C datasets, obtained in two different laboratories, for different human cell types and using different restriction enzymes. We first determined the 3D genome organization in topological domains (TADs) using TopDom algorithm. Then, for each of the 449 diseases considered, the potential over-representation of its associated SNPs in TAD borders (overall covering between about 300 to 500 Mb) has been assessed using a hypergeometric test (see Methods).

**Fig. 2:**
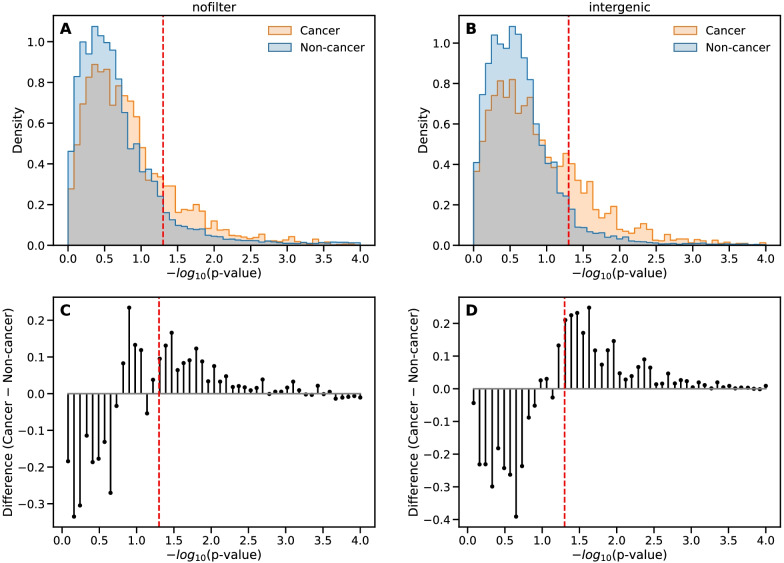
Preferential location in TAD borders of the SNPs associated to a disease. **A** Normalized histogram of enrichment *p*-values (corrected for multiple testing and plotted as [− log_10_ *p*] on the horizontal axis) for cancers (yellow bars) and non-cancer diseases (blue bars, overlap in gray), considering the contribution of all SNPs to a potential TAD-border enrichment. The results have been aggregated over datasets for different cell types from [30] at 10 kb-resolution and all considered values of the window parameter *k* of TopDom algorithm. The two histograms have been normalized separately. The dashed red line indicates the significance threshold at p* = 0.05. **B** Same as (**A**) considering only intergenic daSNPs in the hypergeometric enrichment test. **C** Difference between the cancer histogram, in orange, and the histogram for non-cancer diseases in (**A**), showing that relatively more cancers display a preferential location of their associated SNPs in TAD borders. **D** Same as (**C**) for the histograms in (**B**), showing that the relative dominance of cancers, among diseases displaying TAD-border enrichment, is enhanced when considering only intergenic daSNPs

Results are shown in Fig. 2A in the form of a *p*-value histogram. The *p-*value for a given disease measures the statistical significance of the preferential location of its associated SNPs in TAD borders, or equivalently, the statistical significance of TAD-border enrichment in its associated SNPs. Given the overwhelming number (378) of non-cancer diseases compared to cancers (71) present in the GWAS catalog, and their different etiology, we considered separately cancers and non-cancer diseases.

Our analysis shows that for a fraction of diseases, their associated SNPs are preferentially located in TAD borders, as already observed in a preliminary study [29]. A recent study centered on complex trait heritability consistently put forward a specific status of TAD borders [32]. In our results, the fact that only a fraction of diseases display such a TAD-border enrichment precludes a trivial explanation related to some confounding feature of the TAD borders (e.g., gene density, enhancer location, density of binding sites for architectural proteins, replication origins), that would produce enrichment for all diseases. As an illustration, we give in Additional file 1: File S1 the lists of cancers displaying TAD-border enrichment in their associated SNPs, in the case of IMR90 and NHEK cell lines.

Despite the well-known variability in TAD determination [33–35] (see Methods and Additional file 1: Fig. S2, Additional file 1: Fig. S3), we get a clear statistical result, both for a single value of the parameter *k* (window size) of the TAD caller TopDom, or when aggregating over several values of this parameter.

### TAD-border enrichment in daSNPs is still observed with intergenic SNPs alone

To better interpret the over-representation of daSNPs in TAD borders, we considered specific subsets of daSNPs according to their genomic location: namely daSNPs located in exons (5%), whose variant form has an impact on a protein sequence (coding SNPs) and those, by far more common, located either in introns (55%) or in intergenic regions (40%). We then performed the enrichment analysis considering only intergenic daSNPs. Figure 2B shows that TAD-border enrichment is still observed and even enhanced for intergenic SNPs.

### Preferential location of daSNPs in TAD borders is observed mainly for cancers

The normalized histograms presented in Fig. 2A indicate that the fraction of cancers displaying TAD-border enrichment in daSNPs is larger than the fraction of non-cancer diseases displaying such a preferential location of their associated SNPs. To better evidence this relative predominance of cancers, we present on Fig. 2C the difference between the two histograms. Considering a percentage among cancers is motivated by the small number of cancers (71) in the whole set of diseases (449 EFOs listed in the GWAS). An alternative and equivalent formulation is that the fraction of cancers among the diseases displaying enrichment is larger than the fraction of cancers among the overall set of diseases.

**Fig. 3:**
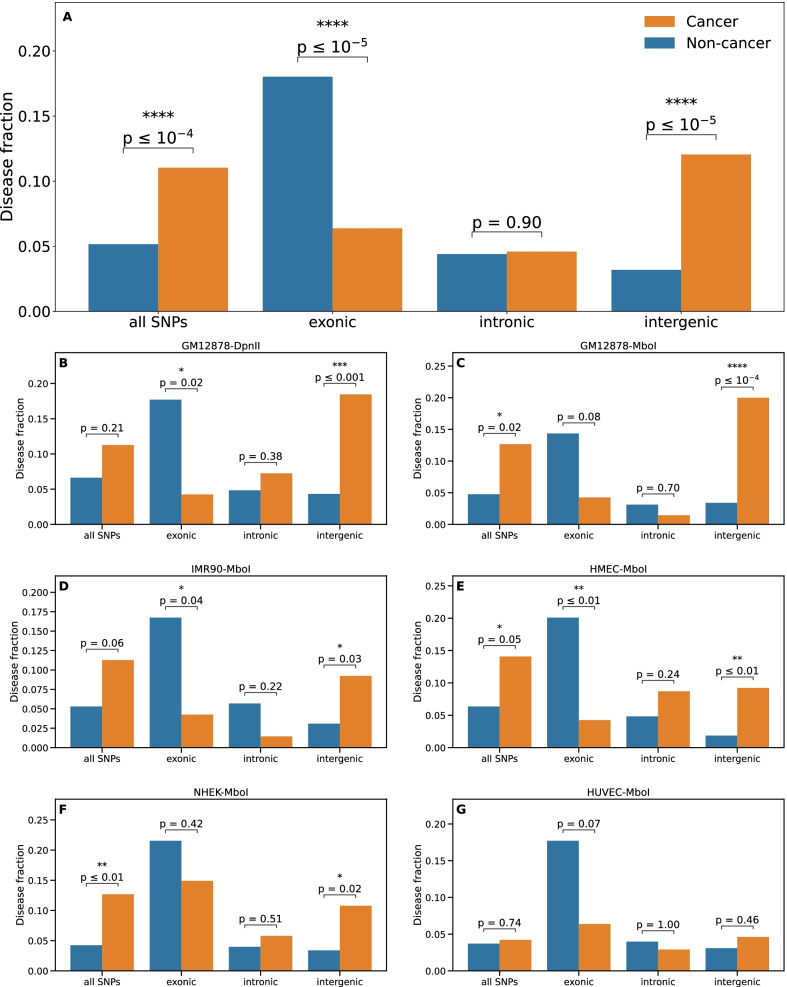
Preferential location of daSNPs in TAD borders occurs relatively more often for cancers. A comparison of the fraction of cancers (orange) and fraction of non-cancer diseases (blue) displaying TAD-border enrichment is presented for various filters on the GWAS catalog: considering all daSNPs, then only exonic, intronic, or intergenic daSNPs. Only diseases displaying enrichment for a majority of values of the TopDom window-size parameter *k* are included (see Methods). **A** Aggregation over five cell types (six datasets) using data at 10 kb-resolution from [30]. **B**–**G** Detailed comparison for each of the six datasets, where the cell type is indicated above each panel, together with the restriction enzyme (DpnII or MboI) used in the Hi-C experiment. Stars indicate when the difference between cancers and non-cancer diseases is statistically significant (Fisher exact test, *p $\le$ 0.05, **p $\le$ 0.01, ****p $\le$ 0.0001). Analyses for data from [19, 36–38] are presented in Additional file 1: Fig. S7

This observation motivated a systematic quantification of the relative predominance of cancers, presented in Fig. 3 together with an assessment of its statistical significance. Similar results have been obtained for the genome-based and the SNP-based null models (see Methods), as expected from the comparison of the enrichment *p*-values obtained with these two null models, presented in Additional file 1: Fig. S4.

We analyzed further the robustness of this relative predominance of cancers among the diseases displaying TAD-border enrichment. We observed that it is sensitive but overall robust with respect to the parameter *k* of the TAD caller (Additional file 1: Fig. S2). This relative predominance of cancers is equally observed before correcting for multiple testing or using a global correction (see Additional file 1: Fig. S5), demonstrating that it is not an artifact of applying the correction separately for cancers and non-cancer diseases.

### The relative predominance of cancers among diseases displaying TAD-border enrichment is enhanced when only intergenic SNPs are considered

We then dissected the contribution of exonic, intronic and intergenic SNPs to TAD-border enrichment. Mainly, the relative predominance of cancers among diseases displaying TAD-border enrichment in intergenic daSNP is confirmed and even enhanced compared to the quantification involving all types of daSNPs (Figs. 2D, 3A). The same shift is observed for both null models (data not shown, but see Additional file 1: Fig. S4), and it is robust with respect to the type of multiple-correction adopted (see Additional file 1: Fig. S6).

That exonic SNPs also contribute to TAD-border enrichment in daSNPs is not surprising nor contradictory, as TAD borders are known to host active genes [19, 39]. We actually observe a stronger and unexpected feature, namely a relative dominance of non-cancer diseases when restricting to exonic daSNPs. However, the low number (a few units) of exonic daSNPs for most diseases brings statistics to a limit and precludes elaborating too much on this observation. In particular, although visible in all cases, the relative dominance of non-cancer diseases reaches statistical significance only in the aggregated data. This observation nevertheless confirms the specificity of cancers, compared to non-cancer diseases, regarding the role of TADs in the associated genetic susceptibility.

### Different enrichments are observed in embryonic stem cells (hESC)

We consistently observe the above-described results for most cell types, as shown in Fig. 3B–G and Additional file 1: Fig. S7. The relative dominance of cancers among diseases displaying TAD-border enrichment in their associated intergenic SNPs largely reaches statistical significance, with a few notable exceptions: umbilical vein cells (HUVEC, Fig. 3G), embryonic stem cells (hESC, Additional file 1: Fig. S7A, E), H1-derived cells (H1_ME, H1_NP, H1_TB, Additional file 1: Fig. S7F, G, H).

**Fig. 4:**
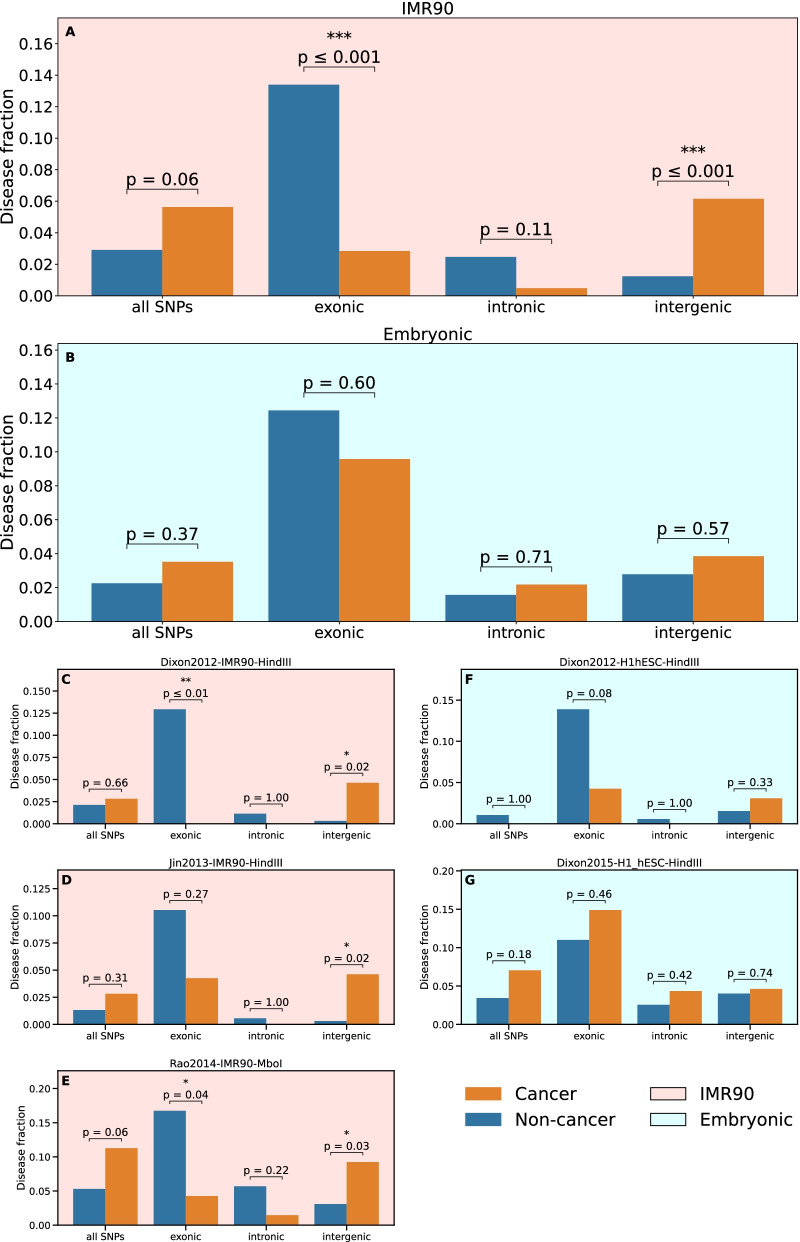
Comparison of enrichment results for IMR90 cells and embryonic cells. Same as Fig. 3, now comparing three datasets for IMR90 cell line (left column, panels **C**, **D** and **E**) with two datasets for embryonic stem cells (hESC, panels **F** and **G**). The two panels at the top display the results aggregated over the individual datasets, for IMR90 cells (pink background, panel **A**) and embryonic stem cells (blue background, panel B), respectively

We further assess the different enrichment results between embryonic stem cells and differentiated cell types in a systematic comparison of hESC and IMR90 cell lines, for which several datasets from different studies are available, Fig. 4. Consistent results are obtained for the two datasets obtained in hESC (Fig. 4, blue panels), and the three datasets for IMR90 (Fig. 4, pink panels), respectively. These results can be extracted more clearly by an aggregation over datasets for the same cell type (Fig. 4, top panels). The comparison emphasizes the peculiarity of hESC, with no relative predominance of cancers among diseases displaying TAD-border enrichment in intergenic daSNPs. In H1 hESC and derived cells at early developmental stage (H1_ME, H1_NP, H1_TB), 3D genome partitioning in TADs is not significantly related to the location of cancer risk loci. Consistently, H1_MS cell line differs from the other H1-derived cell lines both in our analysis and in the original study [38], which has evidenced a genome-wide evolution of TAD structure in this series of H1-derived cell lines following their progressive differentiation. We also observe that HUVEC line has the same signature as hESC, with no cancer-specific features in the location of at-risk SNPs with respect to the TAD structure present in this cell line.

Our analysis thus suggests that TAD-border contribution to the genetic risk of cancers is not observable in HUVEC, hESC and derived cells at early developmental stage (H1_ME, H1_NP, H1_TB) possibly due to their different (not fully mature) TAD structure [38, 40].

### SNP-based diseasome analysis shows that TAD-border enrichment features are not due to a few daSNPs common to several diseases

**Fig. 5:**
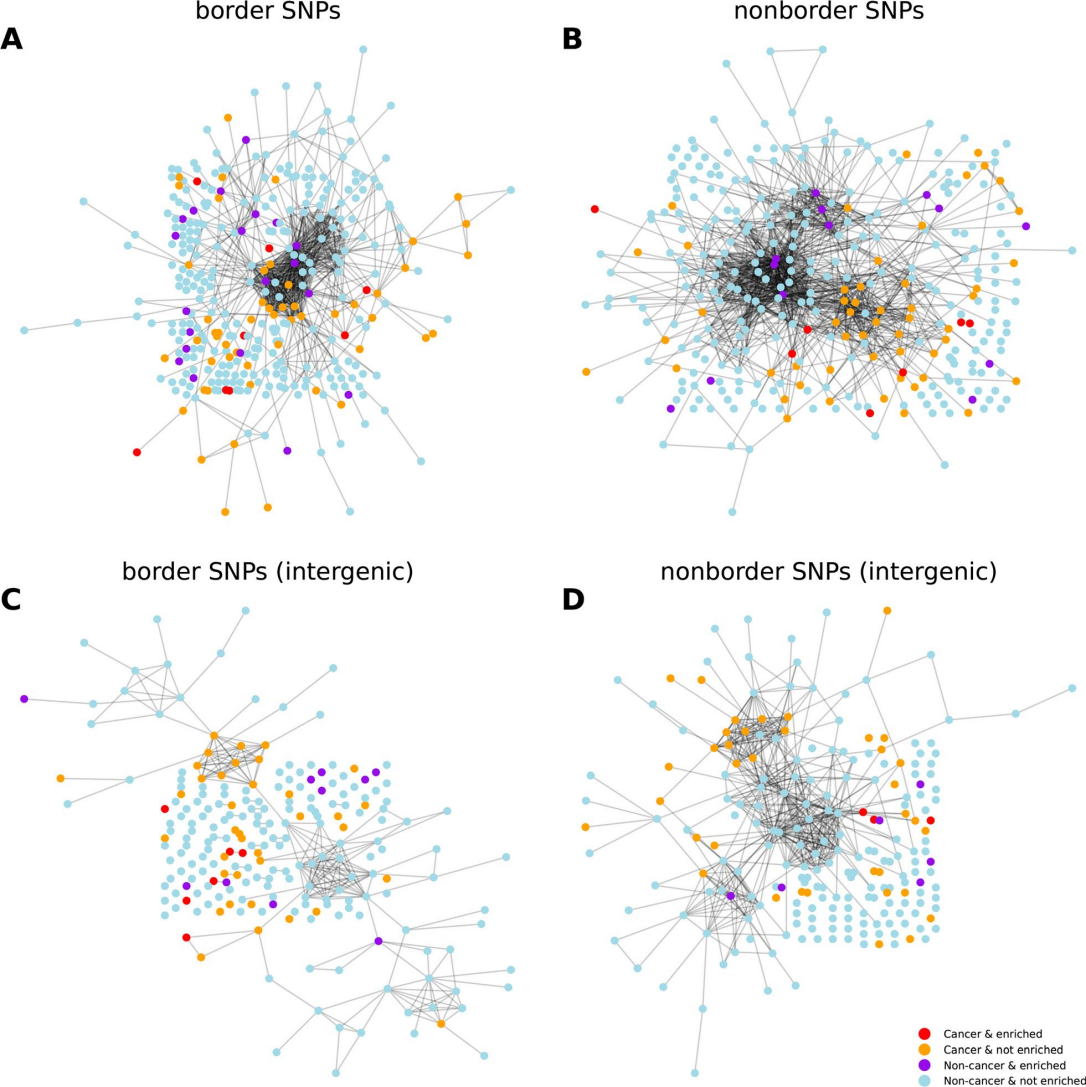
Clustering of cancers and non-cancer diseases sharing daSNPs. In this network representation, red (resp. violet) nodes correspond to cancers (resp. non-cancer diseases) displaying TAD-border enrichment in daSNPs, orange (resp. light blue) nodes to other cancers (resp. other non-cancer diseases). An edge is drawn between two diseases when they share an associated SNP **A** located in a TAD border for a majority of values of the window parameter *k *(border SNPs, see Methods) or **B** belonging to the complementary set (non-border SNPs). **C**, **D** Same as (**A**, **B**) when considering only intergenic SNPs. The four networks have been visualized using *NetworkX* Python package. Underlying Hi-C data from [30], IMR90 cell type, 10 kb-resolution

Among the disease-associated SNPs listed in the GWAS catalog, about 14% are actually associated with several diseases. To analyze the influence of such events on our results, we devise a network representation, where the nodes of the network are diseases (coloring differently cancers and non-cancer diseases) and a link is drawn between two diseases when they share at least one associated SNP. This is nothing but the SNP-based analog of the diseasome networks introduced in [41], in which a link is drawn between two diseases when they share a related gene. We then compared the networks obtained when a link is drawn only when the diseases share a border SNP, i.e., a SNP located in a TAD border for a majority of values of the window parameter (Fig. 5A), when the diseases share a SNP belonging to the complementary set (non-border SNPs, Fig. 5B), then with an additional filter keeping only intergenic SNPs (Fig. 5C, D).

We observe on Fig. 5 that cancers share SNPs preferentially with other cancers, corresponding in the network language to an assortative clustering of cancer nodes. Similar clustering properties are visually observed in the networks based on border SNPs or intergenic border SNPs.

As the visual inspection of the networks can be misleading, we quantified for various node subsets the statistical enrichment of links of each induced subgraph. For this purpose, we used an indicator termed the *network coherence* [42, 43]. Network coherence basically assesses whether the nodes in the set under consideration are more (or less) connected to each other than expected at random. It is defined as a z-score (see Methods) so as to get absolute values than can be used for comparing networks. A random subgraph would have a vanishing network coherence, while a positive value indicates a significantly higher number of internal links compared to random sets of the same size. Additional file 1: Table S1 lists all network coherences for all four networks and all six node types: cancer or non-cancer, displaying TAD-border enrichment (termed in short ‘enriched’) or not.

For non-cancer diseases, the enrichment status makes a difference: The passage from not-enriched to enriched leads to a change of sign (from negative to positive) in the network coherence (from − 1.50 to + 2.09, in the case of border SNPs), suggesting a more important contribution of specific shared SNPs to TAD-border enrichment in the class of non-cancer diseases.

For cancers, the enrichment status does not make a difference: Both subsets of cancers (not-enriched and enriched) have a high network coherence (+ 3.20 and + 2.94, in the case of border SNPs) and, hence, qualitatively speaking, display similar overlaps among SNP sets, either border SNPs or non-border SNPs. The presence of SNPs associated with multiple cancers therefore has a similar impact on our analysis both for cancers displaying TAD-border enrichment and for other cancers. Moreover, the effect becomes weaker when imposing the additional filter of intergenic SNPs. Therefore, our statistical observations regarding TAD-border enrichment in cancer-associated SNPs do not arise from a few shared border SNPs but actually from their preferential location in TAD borders.

## Discussion

We have explored the notion of genetic risk to a disease in the context of the recently acquired knowledge about 3D genome organization, specifically the genome partitioning into topological domains (TADs). We have provided statistical evidence that for some diseases, mostly cancers, associated SNPs are preferentially located in TAD borders. Cancers are relatively more frequent among these diseases, and this relative predominance is enhanced when considering only intergenic SNPs. Network analysis demonstrates that these results are not due to a small number of SNPs common to several diseases.

The fact that the associated SNP is not necessarily the causal variant and may be only a marker related to the causal variation through linkage disequilibrium (LD) does not affect significantly our conclusion: The correlated variations would still be located in the neighborhood of the corresponding TAD border, whose size (20 kb) is often larger than or comparable to the range of strong LD [44, 45]. Moreover, LD has been partly eliminated by the selection of SNP arrays used in GWAS and manual curation in the GWAS catalog. We checked these features by computing, for each disease, the number of pairs of SNPs located at a distance closer than a given threshold, of values ranging from 10 kb to 1 Mb (Additional file 1: Table S2). The resulting low numbers confirm that LD can only be a minor contribution in the interpretation of our observations. In any case, a wider genetic variation (more extended along the genome than a single point mutation, as would follow from linkage disequilibrium) or a correlated genetic variation across a TAD border [45] would even make more plausible an involvement of an architectural change including the TAD border in the risk of developing the disease.

While TAD organization in cancer cells is globally largely intact, many studies have shown that chromatin architecture can be disrupted in cancer by changes in TAD boundaries due to the vast genetic alterations, including copy number variation, mutations, translocations that accompany cancer development and progression (reviewed, e.g., in [28]). Such disruptions lead to aberrant gene expression within the affected TADs. However, very little is known about how genetic variations associated with cancer susceptibility are favoring cancer development in healthy individuals (i.e., before cancer development) and how they impact 3D genome organization. Using capture Hi-C approaches, it was shown that GWAS SNP variants associated with cancer in breast [46], prostate [47] and in colorectal tissues [48] affect long-range chromatin interactions. These pioneer studies suggested that such genetic variants drive altered expression of certain oncogenes and tumor suppression genes, but their impact was so far restricted to chromatin loop organization within TADs. Our results suggest a different mechanism of genetic risk for cancers and non-cancer diseases. Specifically, cancers might be promoted by a joint mis-regulation of oncogenes through a weakening of TAD borders, while non-cancer diseases would rather be favored by local mis-regulations of specific genes (and their cascading consequences).

The weaker relationship between genetic risk loci and TAD borders observed in hESC is consistent with the experimental evidence in mammals of a progressive maturation of the internal structure of TADs, with the establishment of additional enhancer-promoter interactions and further sub-TAD structures during cell differentiation [38, 40]. In other cell lines investigated, TAD-border enrichment in intergenic daSNPs discriminates cancers and non-cancer diseases and suggests an essential difference about the role of genome organization in TADs as regards their genetic risk. These results will now have to be confronted with recent advances in understanding the full complexity of 3D genome organization, its cell-type dependence and its influence on gene regulation [49, 50].

## Conclusions

Our investigation demonstrates a link between genetic risk and 3D genome partitioning into topologically insulated domains. A genetic variation located in TAD borders may weaken the insulation of adjacent TADs, which prevents spurious interactions between genes and enhancers located in adjacent TADs. The larger frequency of cancers among these diseases supports the importance of TAD border weakening in a subset of cancers and emphasizes the different type of gene mis-regulation involved in cancer etiology. A cancer generally involves the malfunction of numerous genes, which is more readily achieved by the extended deregulations induced by weakening of a single TAD border rather than affecting each gene individually. TAD disruption induced by somatic mutations has been observed in cancers [27], and our results suggest that an at-risk variant SNP (and its correlated variations) may act as a head start.

Our results offer the proof-of-concept of a novel criterion for filtering SNPs according to their 3D genomic location, and identifying especially relevant associations, i.e., SNP prioritization. Our study opens a new research avenue in the personalized diagnosis of genetic risk, based on the interplay between 3D genome organization and the location of at-risk SNPs. Dissecting the functional correlates of the preferential location of risk loci in TAD borders now challenges experimental studies. Various experiments, including genome editing to monitor the allelic form of specific loci and chromosome conformation capture techniques, could bring a mechanistic support to this novel statistical evidence of a link between 3D genome organization and the risk of developing certain complex diseases. Our analysis there provides a guideline for experimental studies, in suggesting candidate loci where the mechanisms underlying the genetic risk may involve the effect of the genetic variation on the 3D genome structure.

## Methods

### Disease-associated SNPs (daSNPs)

We used the version v1.0.2-associations_e94_r2018-09-30 of the *GWAS catalog*: www.ebi.ac.uk/gwas/ [51]. We extracted all SNP entries associated with a disease EFO term (*Experimental Factor Ontology*), overall 449 EFO terms, distinguishing 71 cancers and 378 non-cancer diseases. Specifically, diseases were found by selecting all traits which fall into the disease subtree (EFO_0000408) from the EFO ontology, and subsequently, cancers are separated from non-cancer diseases by using the cancer subtree (EFO_0000311).

A SNP can be associated with a disease multiple times in the GWAS catalog when it was found in distinct studies; we ignored this multiplicity by dropping duplicates of its identifier *snpId.*

We classified the resulting 21,183 daSNPs into intergenic (40%), intronic (55%) or exonic (5%) type according to its parent category in *The Sequence Ontology* database, (http://www.sequenceontology.org), version 2015-11-24 [52].We then identified for each disease the subset of its associated intergenic SNPs (on average 47 daSNPs and 18 intergenic daSNPs for both cancer and non-cancer diseases).

### Hi-C data

We used the *Cooler* Hi-C database [53] at ftp://cooler.csail.mit.edu/coolers, which provides published Hi-C data files in the.cool format, at 10 kb-resolution (bin size). Throughout our study, genomic coordinates refer to the hg19 genome version adopted in this database. We present in the main text results obtained with high-resolution data from E. Lieberman-Aiden’s laboratory [30] for the five native and non-cancerous cell lines available, namely GM12878 (human lymphoblastoid cell line, data obtained with MboI or DpnII restriction enzyme), IMR90 (fetal lung fibroblasts of Caucasian origin), HMEC (human mammary epithelial cells), NHEK (normal human epidermal keratinocytes) and HUVEC (human umbilical vein endothelial cells). We considered only normal cell types (and not cancer cell types) as we are interested in the genetic risk present at birth in all cells and wanted to exclude possible confounding features appearing during cancer development itself. We also investigated datasets from B. Ren’s laboratory [19, 36–38], obtained in pioneering experiments using a lower sequencing depth and an enzyme HindIII producing larger restriction fragments (see Additional file 1: Fig. S8), for several cell types: GM12878 in [37], IMR90 in [19, 36] embryonic stem cells (H1 hESC) in [19, 38], and cell lines derived from H1 hESC in [38], namely mesendoderm (H1_ME), neural progenitors (H1_NP) trophoblast-like cells (H1_TB) and mesenchymal cells (H1_MS). Overall, 15 datasets were examined in our study.

### TAD determination

We determined TAD coordinates using the *TopDom* algorithm [31], applied after a transformation of.cool files into count matrices using https://github.com/open2c/cooler. Its principle is to count the number of contacts in a window sliding along the genome and to locate TAD ends at the minima of this count (see Fig. 1, blue diamonds). Genomic regions having established only very few contacts in the experiment, labeled ‘gap’ by TopDom, were filtered out. The choice of using this algorithm is supported by comparative studies [33–35]. Moreover, TopDom is based on quantifying the topological insulation between adjacent TADs, which is the feature that matters for gene (mis)regulation. In particular, recently evidenced long-range associations between TADs and their higher-order organization [54] will not be considered here.

The TAD caller thus involves a tunable parameter *k*, measuring the half-size (in bins, of length equal to the chosen resolution) of the sliding window. This parameter offers a way to investigate the well-known variability in TAD determination [33–35], as depicted in Additional file 1: Fig. S2. The general trend is that larger numbers of TADs are observed for lower values of *k*, at which substructures are also extracted while only large TADs are extracted by the algorithm at large values of *k*. To overcome this technical variability, we scanned all values of the window size *k* from *k* = 3 to *k* = 20 and adopted two strategies: either to aggregate our observations over these values of *k* (Fig. 2), or to use a more stringent majority rule in further analyses (Figs. 3, 4). Both strategies reduce small-number effects and smooth out TAD variability, overall yielding robust results despite the lack of robustness of the TAD landscape (Additional file 1: Fig. S2 and Additional file 1: Fig. S3).

A discrepancy between TADs determined with TopDom and the visual impression given by the contact map could appear locally (see, e.g., Fig. 1), coming from the following difference: TopDom is based on the insulation of TADs, i.e., the presence of low-density (yellow) zones between the triangles delineating the TADs (see Fig. 1), whereas the alternative understanding of a TAD as a region with an increased density of internal contacts would rather focus on dark red triangles emerging from the background in the Hi-C map.

### TAD borders

We defined *TAD borders* as regions of 20 kb located *inside* the TAD at the limits of this TAD. Their size has been chosen smaller than the median size (23 kb) of a human gene [55].

This definition agrees with a topological characterization of TAD borders as regions across which the contact frequency displays a marked decrease [56] and is consistent with the use of TopDom for calling TADs. It differs from the notion of *TAD boundary*, considered, e.g., in [19, 24] which is the—not always existing—linker region *between* two successive TADs along the genome (not belonging to any TAD), whereas a TAD will always have two borders. TAD borders cover from 8% up to 14% of the genome when TopDom parameter *k* varies, the smallest fraction being observed for *k* = 20 (see Additional file 1: File S2, Additional file 1: Table S3 and, for the variation according to the Hi-C dataset, Additional file 1: Table S4). Additionally, to get an idea of the order of magnitude of the numbers involved, the average number of border SNPs for various subsets of diseases (cancers vs non-cancer diseases, displaying or not TAD-border enrichment), and the broad range in which this number varies due to some outlier extreme values, is given in Additional file 1: File S2, together with some figures about SNPs subcategories (exonic, intronic or intergenic).

### Preferential location of daSNPs in TAD borders (TAD-border enrichment)

For each disease (EFO term), we have tested whether the associated SNPs are located in TAD borders more often than observed by chance, where chance (i.e., the null model in statistical terms) corresponds to the same number of pointwise loci drawn at random in the entire genome. In a second analysis, chance corresponds to the same number of SNPs drawn at random in the entire set of disease-associated SNPs listed in the GWAS catalog. The statistical significance of a preferential location of daSNPs in TAD borders is then assessed by computing a *p*-value for the disease according to a hypergeometric test.

In more detail:

Testing a preferential location in TAD borders of the SNPs associated to a given disease involves the hypergeometric distribution *H (q | N, n, Q)* describing the probability of getting *q* border SNPs when drawing *Q* elements (as many as the number of SNPs associated to the considered disease) at random, without replacement, in the null model. In the *genome-based null model*, *N* is the total number of base pairs in the genome and *n* the part located in TAD borders. In the *SNP-based null model*, close to that considered, e.g., in [57]*, N* is the total number of disease-associated SNPs listed in the GWAS catalog (each counted once, and not including the SNPs associated with non-pathological traits so that the random model is the closest possible to the data) and *n* the part located in TAD borders. The computation for testing TAD-border enrichment is performed at the SNP level, i.e., at the base-pair level: Two SNPs located in the same bin, or in the same border, will be counted as two units. These occurrences are very rare events (see Additional file 1: Table S2). Results presented in the main text were obtained with the more conservative genome-based null model. A comparison with those obtained with the SNP-based null model is presented in Additional file 1: Fig. S4.

The *p*-value for the considered disease, assessing the over-representation of its associated SNPs in TAD borders, is computed as the cumulative distribution function (i.e., the fraction of values larger than or equal to *q*) of this hypergeometric distribution. Given the symmetry property *H (q | N, n, Q)* = *H (q | N, Q, n*) of the hypergeometric distribution, it is equivalent to state that (i) the SNPs associated with the disease are located in a TAD border more often than expected by chance, or (ii) TAD borders contain a SNP associated with this disease more often than expected by chance. We term such a situation *TAD-border enrichment.*

After computing a raw *p-*value for each disease, Benjamini–Hochberg procedure (*multipletests* function with method *fdr_bh* from the *statsmodels* package in Python, www.statsmodels.org/dev/index.html#citation) is applied to obtain *p*-values adjusted for multiple testing, so that the false discovery rate is controlled at level 5% when the adjusted *p*-value is lower than 0.05 [58]. Given the overwhelming number of non-cancer diseases (378) compared to cancers (71) and their different etiology, we investigated separately these two groups of diseases. These two groups are well-defined on biological criteria independently of our enrichment testing, so that correction for multiple testing has been applied separately in each group. Nevertheless, we checked that our main result (TAD-border enrichment in daSNPs for certain diseases and its relatively higher frequency for cancers) remains qualitatively observed when we used a global multiple-testing correction, considering jointly cancers and non-cancer diseases, or even no correction (Additional file 1: Fig. S5 and Additional file 1: Fig. S6)*.*

### Enrichment histograms

Histograms of corrected *p-*values (Fig. 2) are plotted and normalized separately for cancers and non-cancer diseases. The counts have been first aggregated over the six considered datasets from [30] and the values of the window parameter *k* of the TAD caller. In order to get a better display of the core features of the plots, the range of *p*-values has been truncated at log_10_(1/*p*) = 4. A few EFOs, with corrected *p-*values smaller than 0.0001, are thus lying outside the displayed plot. It would be possible to choose a larger bin size or even to draw a smooth histogram, but this would rather dilute the information.

### Comparison of TAD-border enrichment for cancers and non-cancer diseases

Due to the small number of cancers (71) compared to non-cancer diseases (378), we compared the fraction of cancers and the fraction of non-cancer diseases displaying a significant TAD-border enrichment, considering either all their associated SNPs or only a sub-category (exonic, intronic and intergenic). Only diseases displaying a significant enrichment for a majority (more than 50%) of values of the window parameter *k* have been counted. The significance of a difference between the disease fractions has been assessed using Fisher’s exact test. The comparison has been done for each of the six considered datasets (for 5 cell types) from [30], and also after aggregating the disease counts over these datasets.

### Workflow

The different steps described above have been gathered in an easy-to-execute pipeline, using *Snakemake *(https://snakemake.github.io, [59]) unifying the analysis of different datasets. Its rule graph is presented on Additional file 1: Fig. S1. Its code, written in Python, is freely available at https://github.com/kpj/GeneticRiskAndTADs. An order of magnitude of the typical numbers of diseases and SNPs of different categories involved in our analysis is provided in Additional file 1: File S2, Additional file 1: Table S3, Additional file 1: Table S4.

### SNP-based diseasome network and its analysis

We introduced a network representation where nodes are diseases and an edge is drawn between two diseases when they share an associated SNP. Starting from a bipartite network relating diseases and their associated SNPs, this representation is the projection on disease nodes. It is the analog for SNPs of the network relating diseases and their associated genes, known as the diseasome, and its projected version [41]. A filter has been applied on shared SNPs: in the network labeled ‘border SNPs,’ an edge is drawn when the diseases share a SNP lying in a TAD border for a majority of values of the window parameter *k* (underlying Hi-C data from [30], IMR90 cell type). The network labeled ‘non-border SNPs’ involves the complementary set of SNPs. Additionally, the two networks have been re-drawn considering only intergenic SNPs. Non-cancer disease and cancer nodes (and among them, those displaying TAD-border enrichment) were underlined with different colors. Note that, each cancer or each non-cancer disease is associated with an ensemble of border SNPs, of non-border SNPs, of intergenic border SNPs and of intergenic non-border SNPs, and could be present in more than one of the four networks. In Fig. 5, the four networks have been visualized using *NetworkX* Python package. For each network, only diseases (nodes) having an associated SNP of the prescribed type are drawn.

For a quantitative comparison of the four networks regarding their clustering and assortativity properties, we computed an indicator for any subset of nodes, e.g., a group of nodes with the same color. This indicator, called *network coherence*, is defined as the z-score of the number of edges within the subset of nodes, compared to a thousand randomly drawn groups of nodes of the same size [42, 43]. Network coherence thus measures whether the induced subgraph is more densely connected (i.e., contains more links) than expected at random in the original network. As a z-score, it provides an absolute quantification, independent of the overall size of the group, which makes cross-comparisons possible. Choosing a threshold larger than 1 on the number of shared SNPs required to draw an edge does not change qualitatively the results but reduces the number of diseases involved, which brings statistics to a limit. All the results presented in the text were obtained with a threshold equal to 1.

## Supplementary Information

**Additional file 1.** Supplementary Information including: **Fig. S1:** Rule graph of the integrated pipeline. **File S1:** Lists of cancers displaying TAD border enrichment in their associated SNPs. **Fig. S2:** Variation of TADs and TAD borders at varying TopDom parameter k. **Fig. S3:** Variation of TAD and TAD borders across data sources. **Fig. S4:** Comparison of two null models for assessing TAD border enrichment in daSNPs. **Fig. S5:** Multiple testing correction in assessing TAD border enrichment in daSNPs. **Fig. S6:** Multiple testing correction in assessing TAD border enrichment in intergenic daSNPs. **Fig. S7:** TAD border enrichment across data sources. **Table S1:** Values of network coherence for subgraphs in SNP-based diseasome networks. **Table S2:** Analysis of pairwise distance between daSNPs. **Fig. S8:** Restriction fragment size distributions. **File S2:** Typical numbers of diseases and SNPs involved in the analysis. **Table S3:** Genome fraction located in TAD borders. **Table S4:** Genome fraction and number of SNPs in TAD borders for different datasets.

## Data Availability

The datasets supporting the conclusions of this article are available in the Cooler repository: https://github.com/open2c/cooler (datasets gathered in.cool format [53] from published studies [19, 30, 36–38]. The pipeline specifically devised for our study is available at https://github.com/kpj/GeneticRiskAndTADs.

## Abbreviations

3D: 3-Dimensional
GWAS: Genome-wide association studies
hESC: Human embryonic stem cell
Hi-C: Genome-wide chromosome conformation capture
IMR90: Human fetal lung fibroblasts
LD: Linkage disequilibrium
SNP: Single-nucleotide polymorphism
daSNP: Disease-associated SNP
TAD: Topologically associating domain
