# Supplementary material for: Contribution of 3D genome topological domains to genetic risk of cancers: a genome-wide computational study
Source: Hum Genomics. 2022 Jan 11;16:2. doi: 10.1186/s40246-022-00375-2 (PMC8753905; doi:10.1186/s40246-022-00375-2)
Supplement: Supplementary file 1 — Additional file 1. Supplementary Information including: Fig. S1: Rule graph of the integrated pipeline. File S1: Lists of cancers displaying TAD border enrichment in their associated SNPs. Fig. S2: Variation of TADs and TAD borders at varying TopDom parameter k. Fig. S3: Variation of TAD and TAD borders across data sources. Fig. S4: Comparison of two null models for assessing TAD border enrichment in daSNPs. Fig. S5: Multiple testing correction in assessing TAD border enrichment in daSNPs. Fig. S6: Multiple testing correction in assessing TAD border enrichment in intergenic daSNPs. Fig. S7: TAD border enrichment across data sources. Table S1: Values of network coherence for subgraphs in SNP-based diseasome networks. Table S2: Analysis of pairwise distance between daSNPs. Fig. S8: Restriction fragment size distributions. File S2: Typical numbers of diseases and SNPs involved in the analysis. Table S3: Genome fraction located in TAD borders. Table S4: Genome fraction and number of SNPs in TAD borders for different datasets. [file 40246_2022_375_MOESM1_ESM.pdf]

SUPPLEMENTARY MATERIAL

Contribution of 3D genome topological domains to genetic risk of cancers: a genome-wide computational study

Kim Philipp Jablonski, Leopold Carron, Julien Mozziconacci, Thierry Forné, Marc-Thorsten Hütt and Annick Lesne

Contents:

Supplementary Fig 1. Rule graph of the integrated pipeline ……………………………………………................... 2
Supplementary File 1. Lists of cancers displaying TAD border enrichment in their associated SNPs … …….3
SupplementaryFig 2. Variation of TADs and TAD borders at varying TopDom parameter *k*.…………..…..… 5
SupplementaryFig 3. Variation of TAD and TAD borders across data sources……..……………..………..… 6
Supplementary Fig 4. Comparison of two null models for assessing TAD border enrichment in daSN……..... 7
Supplementary Fig 5. Multiple testing correction in assessing TAD border enrichment in daSNPs……......... 8
Supplementary Fig 6. Multiple testing correction in assessing TAD border enrichment in intergenic daSNPs...9
Supplementary Fig 7. TAD border enrichment across data sources……………………………………….… 10
Supplementary Table 1. Values of network coherence for subgraphs in SNP-based diseasome networks…... 11
Supplementary Table 2. Analysis of pairwise distance between daSNPs……………………………..…….12
SupplementaryFig 8. Restriction fragment size distributions……………………………….………..…..… 13
Supplementary File 2. Typical numbers of diseases and SNPs involved in the analysis…………………..….. 14
Supplementary Table 3. Genome fraction located in TAD borders……………………………………...… 14
Supplementary Table 4. Genome fraction and number of SNPs in TAD borders for different datasets….….. 15
References in the Supplementary Material ………..…………………………………………………….… 15

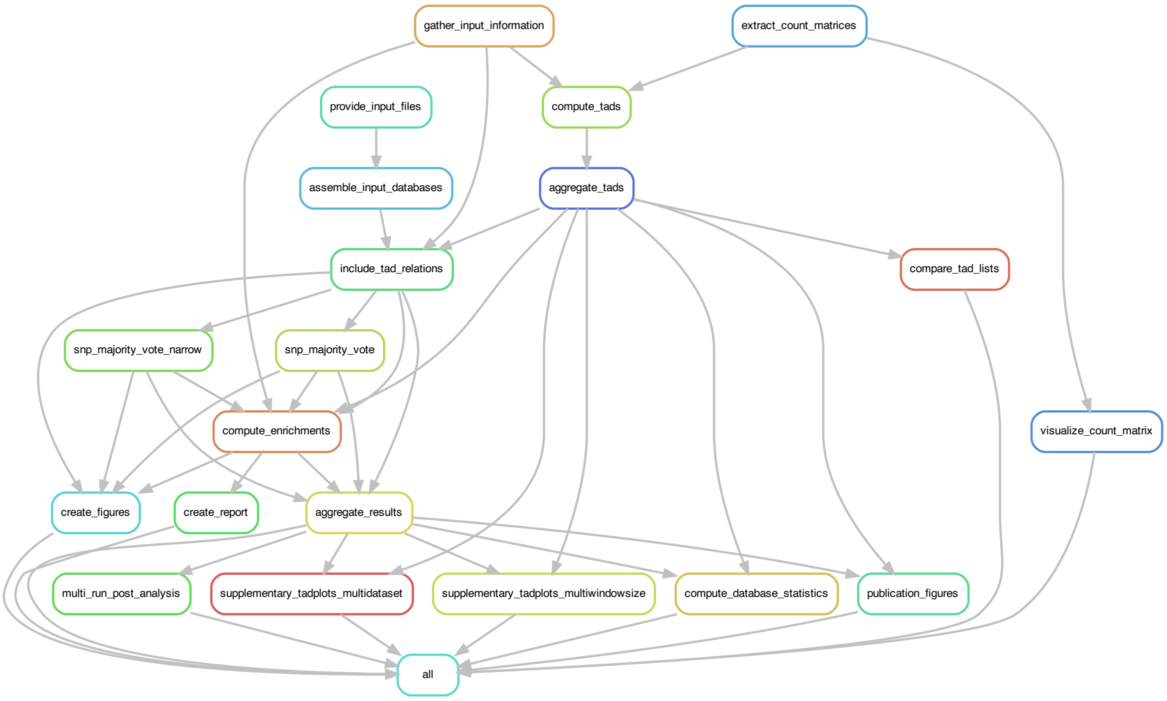

Supplementary Fig 1. Rule graph of the integrated pipeline.

The figure displays an automated drawing of the bioinformatic and statistical pipeline devised for the study. For each disease, the potential TAD border enrichment in disease-associated SNPs is assessed from the associations available in the GWAS catalog and Hi-C data, downloaded from ftp://cooler.csail.mit.edu/coolers. The test involves the determination of TADs using TopDom algorithm [S1] and the computation of the *p*-value quantifying the statistical significance of the enrichment (see *Methods)*. The subsequent steps of the analysis, for instance the aggregation of the results over several values of the parameter *k* of the TAD caller, the plots of enrichment histograms (Fig 2) or percentages (Fig 3), have been integrated in the pipeline, available at: https://github.com/kpj/GeneticRiskAndTADs. The whole analysis can thus be implemented straightforwardly for any Hi-C dataset in .cool format.

Supplementary File 1: Lists of cancers displaying TAD border enrichment in their associated SNPs
We present below two lists obtained using Hi-C data from [S2], at 10kb resolution, for NHEK and IMR90ncell lines, including only cancers displaying TAD border enrichment when border SNPs are defined using a majority rule (i.e. as the SNPs being in a border for a majority of values of the TAD caller parameter *k*). The presence or absence of an entry has a variable degree of robustness, so these lists are provided only to illustrate the outcomes of our analysis. Reliable conclusions for a specific cancer would require a dedicated detailed study.

IMR90 cell line, Hi-C data from [S2]:

EFO_0000094 B-cell acute lymphoblastic leukemia
EFO_0000095 chronic lymphocytic leukemia
EFO_0000096 neoplasm of mature B-cells
EFO_0000174 Ewing sarcoma
EFO_0000178 gastric carcinoma
EFO_0000181 head and neck squamous cell carcinoma
EFO_0000182 hepatocellular carcinoma
EFO_0000183 Hodgkins lymphoma
EFO_0000220 acute lymphoblastic leukemia
EFO_0000292 bladder carcinoma
EFO_0000305 breast carcinoma
EFO_0000311 cancer
EFO_0000326 central nervous system cancer
EFO_0000389 cutaneous melanoma
EFO_0000403 diffuse large B-cell lymphoma
EFO_0000478 esophageal adenocarcinoma
EFO_0000503 gastric adenocarcinoma
EFO_0000519 glioblastoma multiforme
EFO_0000571 lung adenocarcinoma
EFO_0000574 lymphoma
EFO_0000637 osteosarcoma
EFO_0000641 papillary thyroid carcinoma
EFO_0000681 renal cell carcinoma
EFO_0000702 small cell lung carcinoma
EFO_0000707 squamous cell carcinoma
EFO_0000708 squamous cell lung carcinoma
EFO_0000756 melanoma
EFO_0001061 cervical carcinoma
EFO_0001071 lung carcinoma
EFO_0001075 ovarian carcinoma
EFO_0001378 multiple myeloma
EFO_0001663 prostate carcinoma
EFO_0002618 pancreatic carcinoma
EFO_0003060 non-small cell lung carcinoma
EFO_0004193 basal cell carcinoma
EFO_0004708 nodular sclerosis Hodgkin lymphoma
EFO_0005088 testicular carcinoma
EFO_0005220 pulmonary neuroendocrine tumor
EFO_0005221 cholangiocarcinoma
EFO_0005543 glioma
EFO_0005570 oral cavity cancer
EFO_0005577 pharynx cancer
EFO_0005842 colorectal cancer
EFO_0005922 esophageal squamous cell carcinoma
EFO_0006352 laryngeal squamous cell carcinoma
EFO_1000218 Digestive System Carcinoma
EFO_1000630 marginal zone B-cell lymphoma
EFO_1000649 estrogen-receptor positive breast cancer
EFO_1000650 estrogen-receptor negative breast cancer
EFO_1000654 childhood cancer
EFO_1000657 rectum cancer
EFO_1001480 metastatic colorectal cancer
EFO_1001512 endometrial carcinoma
EFO_1001514 endometrial endometrioid carcinoma
EFO_1001515 ovarian endometrioid carcinoma
EFO_1001516 ovarian serous carcinoma
EFO_1001927 cutaneous squamous cell carcinoma
EFO_1001931 oropharynx cancer

EFO_1002017 differentiated thyroid carcinoma

NHEK cell line, Hi-C data from [S2], majority rule over *k*

EFO_0000094 B-cell acute lymphoblastic leukemia
EFO_0000095 chronic lymphocytic leukemia
EFO_0000096 neoplasm of mature B-cells
EFO_0000174 Ewing sarcoma
EFO_0000178 gastric carcinoma
EFO_0000181 head and neck squamous cell carcinoma
EFO_0000182 hepatocellular carcinoma
EFO_0000183 Hodgkins lymphoma
EFO_0000203 monoclonal gammopathy
EFO_0000220 acute lymphoblastic leukemia
EFO_0000292 bladder carcinoma
EFO_0000305 breast carcinoma
EFO_0000311 cancer
EFO_0000326 central nervous system cancer
EFO_0000389 cutaneous melanoma
EFO_0000403 diffuse large B-cell lymphoma
EFO_0000478 esophageal adenocarcinoma
EFO_0000503 gastric adenocarcinoma
EFO_0000519 glioblastoma multiforme
EFO_0000553 invasive lobular carcinoma
EFO_0000571 lung adenocarcinoma
EFO_0000574 lymphoma
EFO_0000637 osteosarcoma
EFO_0000641 papillary thyroid carcinoma
EFO_0000681 renal cell carcinoma
EFO_0000702 small cell lung carcinoma
EFO_0000707 squamous cell carcinoma
EFO_0000708 squamous cell lung carcinoma
EFO_0000756 melanoma
EFO_0001061 cervical carcinoma
EFO_0001071 lung carcinoma
EFO_0001075 ovarian carcinoma
EFO_0001378 multiple myeloma
EFO_0001663 prostate carcinoma
EFO_0002618 pancreatic carcinoma
EFO_0003060 non-small cell lung carcinoma
EFO_0004193 basal cell carcinoma
EFO_0004708 nodular sclerosis Hodgkin lymphoma
EFO_0005088 testicular carcinoma
EFO_0005221 cholangiocarcinoma
EFO_0005537 triple-negative breast cancer
EFO_0005543 glioma
EFO_0005842 colorectal cancer
EFO_0005922 esophageal squamous cell carcinoma
EFO_0006352 laryngeal squamous cell carcinoma
EFO_0006462 ovarian mucinous adenocarcinoma
EFO_1000218 Digestive System Carcinoma
EFO_1000630 marginal zone B-cell lymphoma
EFO_1000649 estrogen-receptor positive breast cancer
EFO_1000650 estrogen-receptor negative breast cancer
EFO_1000654 childhood cancer
EFO_1000657 rectum cancer
EFO_1001480 metastatic colorectal cancer
EFO_1001512 endometrial carcinoma
EFO_1001514 endometrial endometrioid carcinoma
EFO_1001515 ovarian endometrioid carcinoma
EFO_1001516 ovarian serous carcinoma
EFO_1001927 cutaneous squamous cell carcinoma
EFO_1001931 oropharynx cancer
EFO_1002017 differentiated thyroid carcinoma

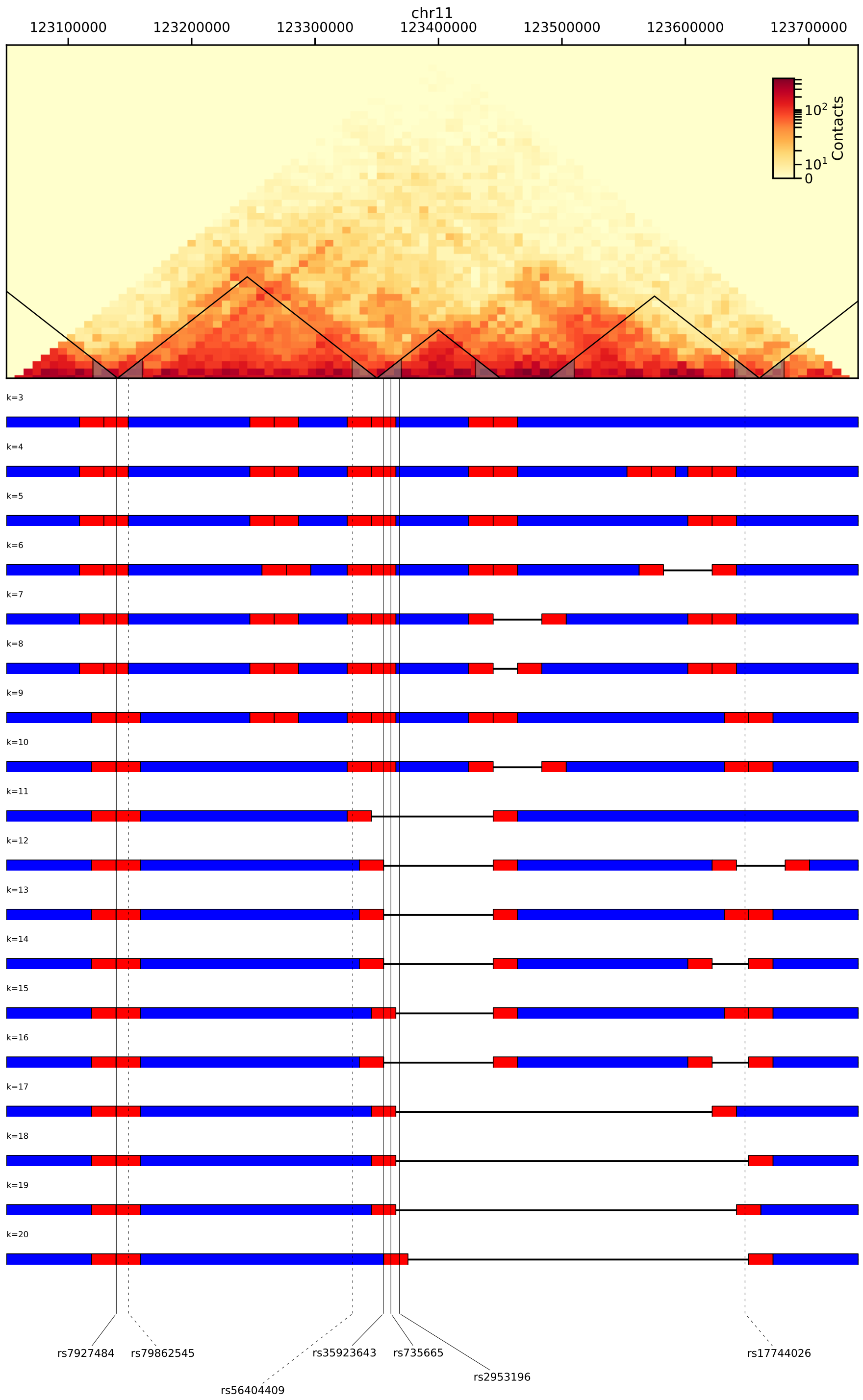

Supplementary Fig 2. Variation of TADs and TAD borders at varying TopDom parameter *k*.
The top panel displays the Hi-C contact matrix as a heat map (see the color bar, the redder the more contacts), here for a region of chr11 (chr11: 123050000-123750000, hg19 coordinates, embedding the region in Fig 1), drawn from Hi-C data published in [S2], for IMR90 cell type, at 10kb-resolution. TADs determined with TopDom, for a window size *k*=10, are underlined together with their internal 20kb-borders. Vertical lines pinpoint SNPs located in a TAD border (full line for cancer-associated SNPs, dashed lines for SNPs associated with non-cancer diseases). TADs obtained at increasing value of *k*, from *k*=3 to *k*=20, are schematically displayed in the lines below (blue: TAD body, red: TAD border, value of k indicated above each line at its left end).

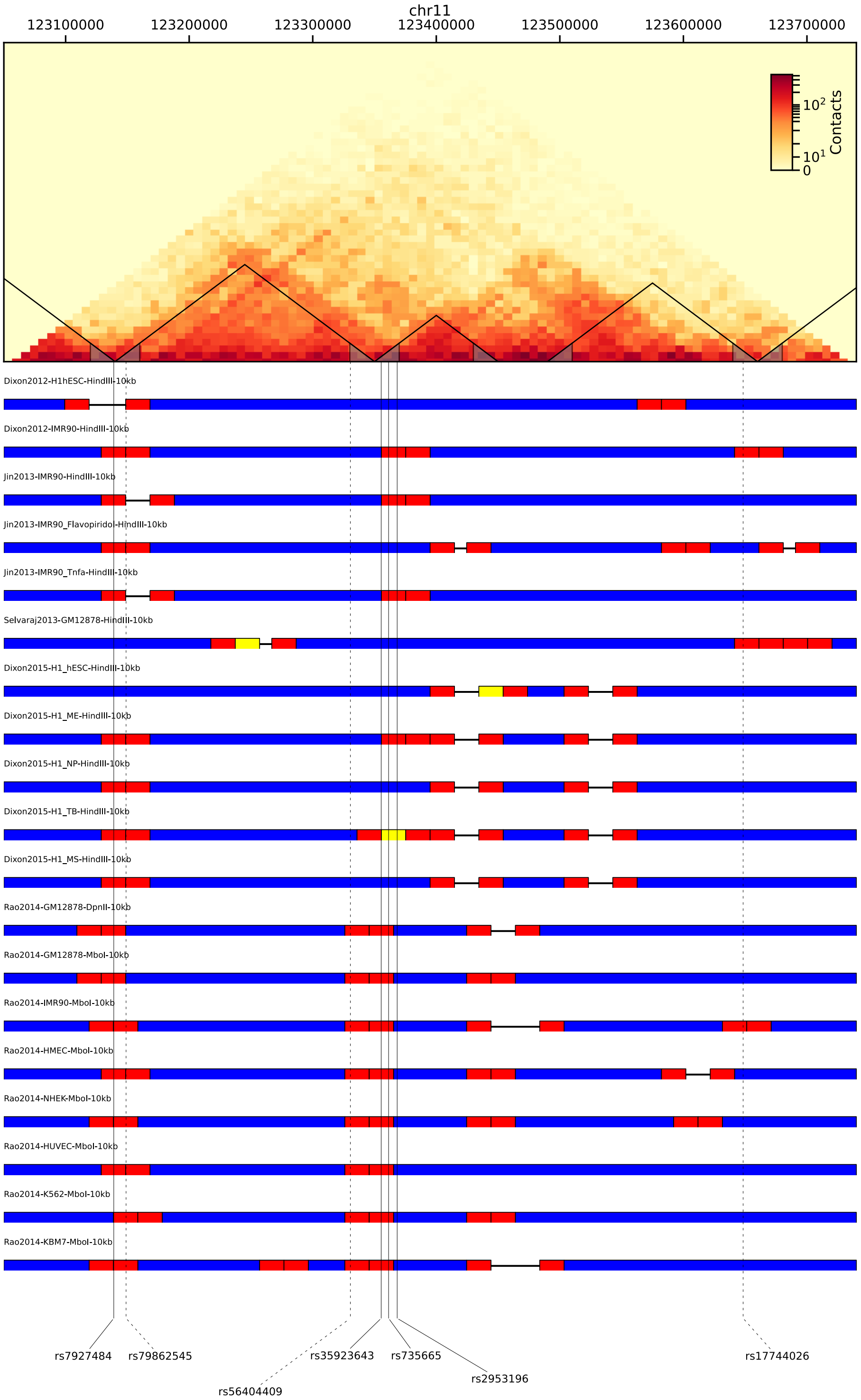

Supplementary Fig 3. Variation of TAD and TAD borders across data sources.
Same as the previous Supplementary Fig 2, now for different data sources, indicated above each line, at a fixed value *k*=10 of TopDom window parameter (blue: TAD body, red: TAD border defined as the end region of size 20kb within the TAD, yellow: situations where a TAD is too short and its two borders overlap). The Hi-C map is based on Hi-C data from [S2], IMR90 cell type, at 10kb-resolution, for the region chr11: 123050000-123750000, hg19 coordinates (same region as in Supplementary Fig 2 and embedding the region in Fig 1).

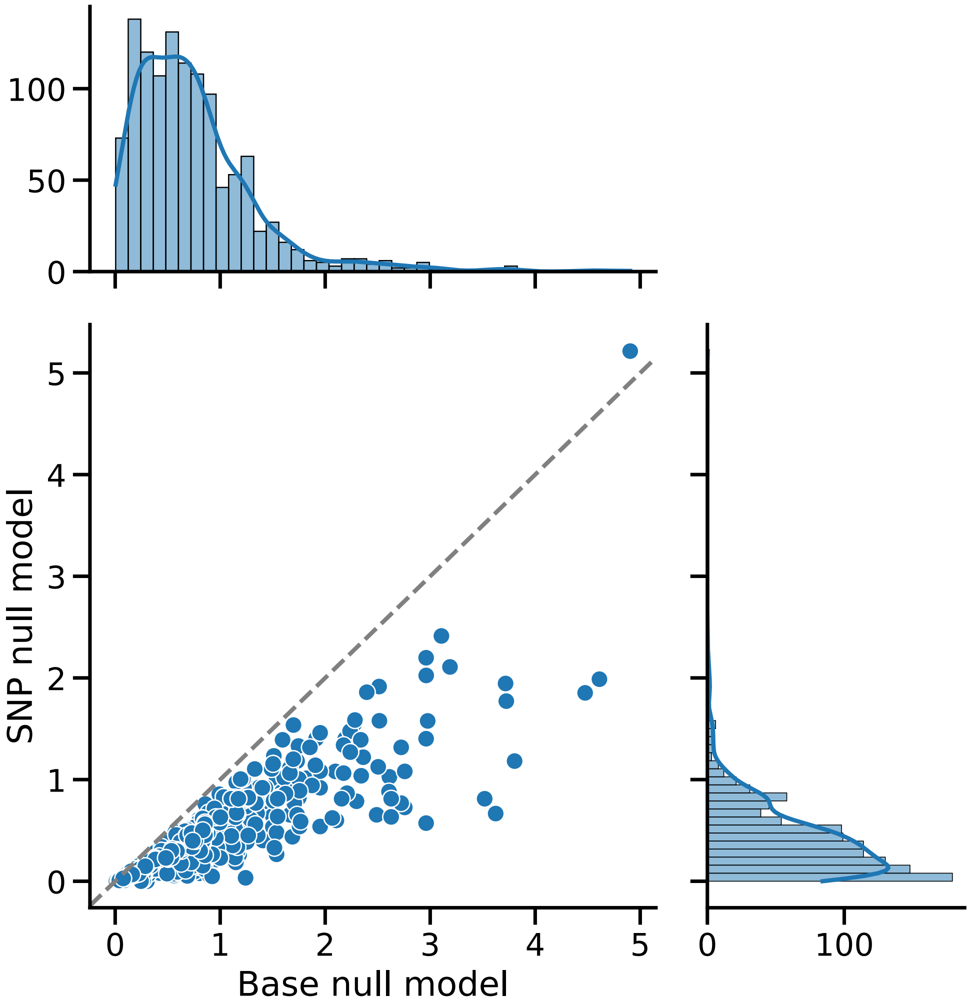

Supplementary Fig 4. Comparison of two null models for assessing TAD border enrichment in daSNPs. The figure presents a scatter plot of the enrichment statistical significance [-log(*p*-value)], one dot per disease, for two different null models (see *Methods*) and, on the right and top respectively, the *p*-value distribution over all diseases for each null model. The first null model (horizontal axis in the scatter plot, distribution on top) is defined for each disease as a homogeneous distribution of the associated SNPs along the genome, at the base pair level, whereas the second one (vertical axis in the scatter plot, distribution on the right) is defined for each disease as a homogeneous distribution of the associated SNPs within the ensemble of all disease-associated SNPs gathered from the GWAS catalog (not including SNPs associated with non-pathological traits). The *p*-value assessing for each disease the over-representation of its associated SNPs in TAD borders is computed using the cumulative hypergeometric distribution, then corrected for multiple testing using Benjamini-Hochberg procedure [S3] separately for cancers and non-cancer diseases (see *Methods)*. The correlation coefficient between the results obtained with each null model is 0.92. Hi-C data from [S2], IMR90 cell type, 10kb resolution; TAD determination with TopDom window parameter value k=10.

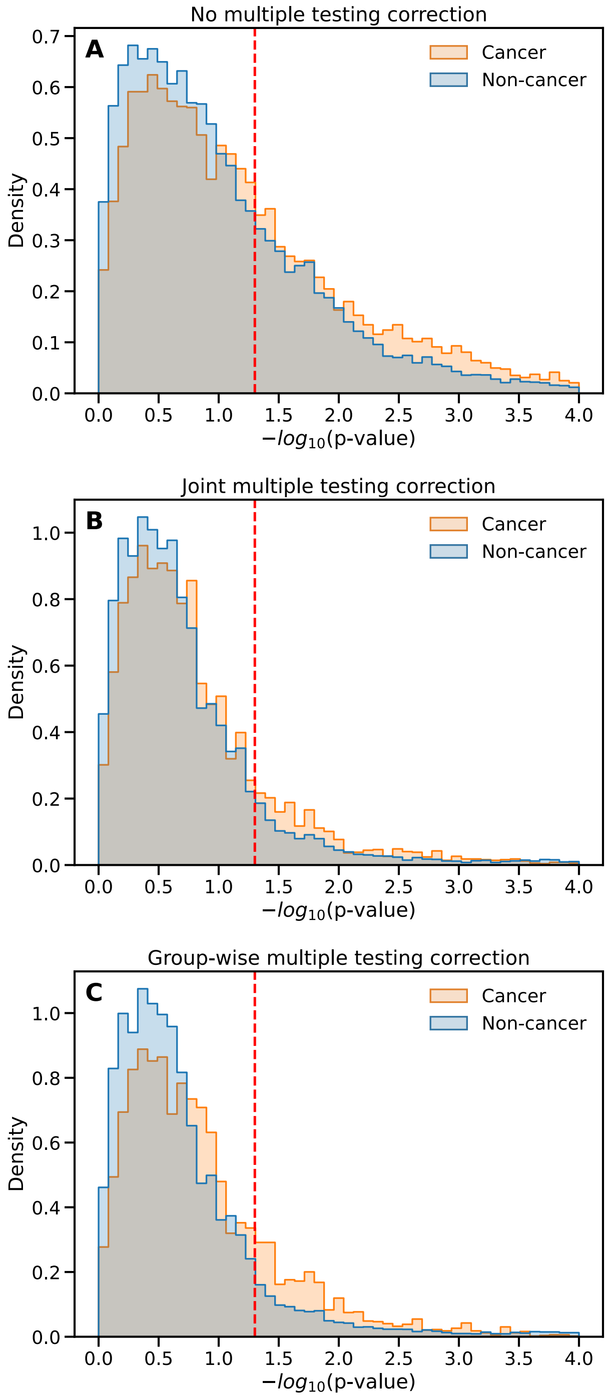

Supplementary Fig 5. Multiple testing correction in assessing TAD border enrichment in daSNPs. Histograms of [-log(*p*-value)] for cancers (orange) and non-cancer diseases (blue, overlap in grey) (A) without multiple-testing correction; (B) with a correction applied to all diseases jointly; (C) with a correction applied separately to cancers and non-cancer diseases (group-wise correction, see Fig 2). In cases B and C, the correction followed Benjamini-Hochberg procedure [S3] controlling the false discovery rate. The significance threshold at 5% is indicated by the dashed red line. Histograms are normalized separately for cancers and non-cancer diseases. Same underlying Hi-C data and setting as in Fig 2.

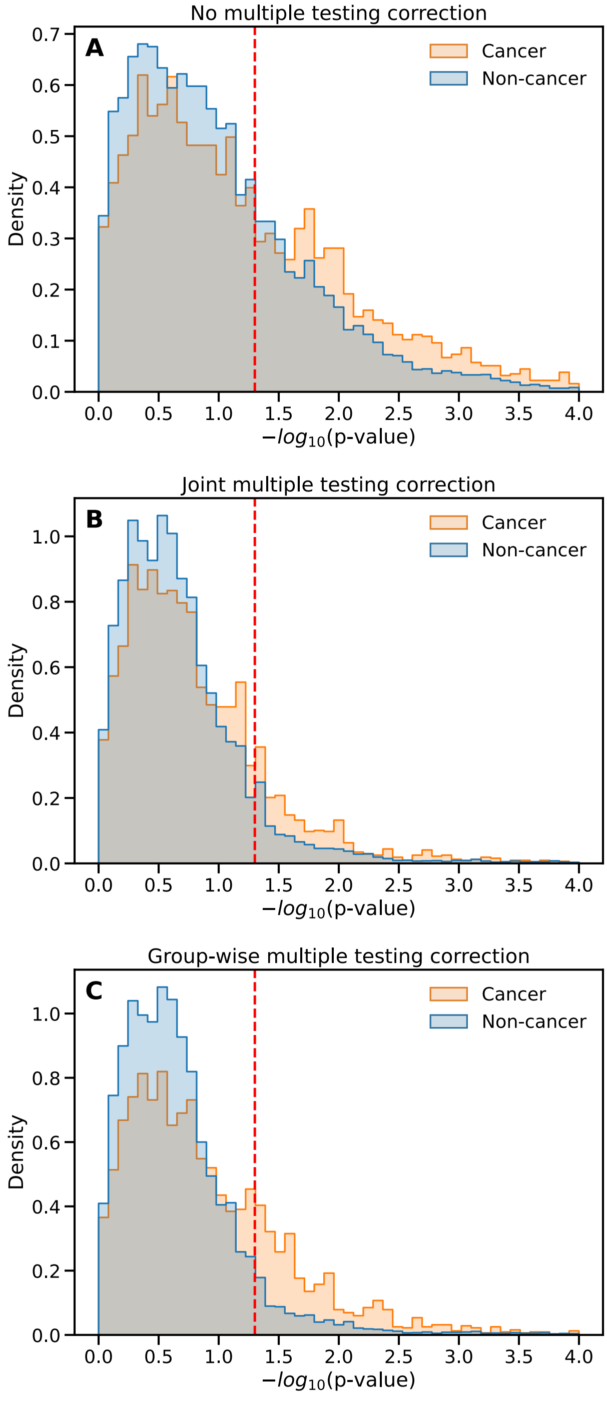

Supplementary Fig 6. Multiple testing correction in assessing TAD border enrichment in intergenic daSNPs. Same as Supplementary Fig 5 but considering intergenic SNPs only. The figure displays the normalized histograms of [-log(*p*-value)] for cancers (orange) and non-cancer diseases (blue, overlap in grey) and (A) without multiple-testing correction; (B) with a correction applied to all diseases jointly; (C) with a correction applied separately to cancers and non-cancer diseases (group-wise correction, see Fig 2).

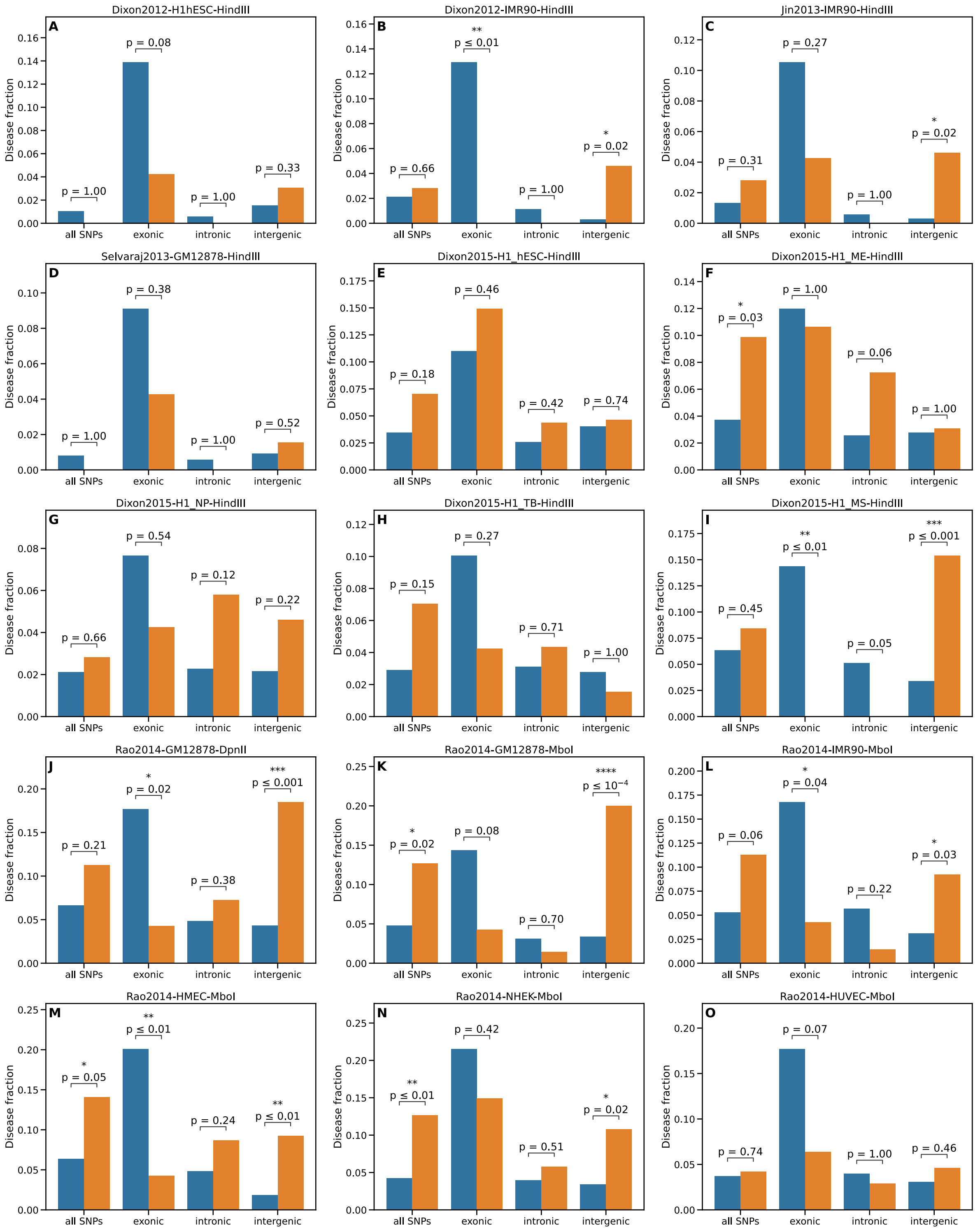

Supplementary Fig 7. TAD border enrichment across data sources.
The relative dominance of cancers among the diseases displaying a preferential location of their associated SNPs in TAD borders is investigated across Hi-C datasets (same setting as in Fig 3). The datasets were obtained in different laboratories (main ordering of the panels), or/and with different restriction enzymes (HindIII, MboI or DpnII, as indicated in the caption, see next page Supplementary Fig 8) or/and in different cell types: human embryonic stem cells (H1 hESC) and derived cell lines: mesendoderm (H1_ME), neural progenitors (H1_NP), trophoblast-like cells (H1_TB) and mesenchymal cells (H1_MS); human lymphoblastoid cell line (GM12878); fetal lung fibroblasts of Caucasian origin (IMR90); human mammary epithelial cells (HMEC); normal human epidermal keratinocytes (NHEK). human umbilical vein endothelial cells (HUVEC) [S2, S4-S7].

diseasome network	cancer	non-cancer	enriched cancer	enriched non-cancer	not enriched cancer	not enriched non-cancer
‘border’	**4.02**	**-1.26**	**2.94**	**2.09**	**3.20**	**-1.50**
‘border intergenic’	**2.75**	**-0.15**	**4.24**	**4.04**	**1.67**	**-0.60**
‘non-border’	**5.02**	**-1.59**	**3.45**	**3.55**	**4.56**	**-2.92**
‘non-border intergenic’	**2.62**	**0.09**	**2.29**	**4.85**	**2.61**	**-1.29**

Supplementary Table 1: Values of network coherence for subgraphs in SNP-based diseasome networks. Nodes in the diseasomes represent diseases, distinguishing cancers or non-cancer diseases. Four SNP-based diseasome networks have been constructed, depending on the meaning of an edge between two nodes. In the network labelled ‘border’, an edge is drawn between two diseases when they share at least one border SNP, i.e. a SNP located in a TAD border for a majority of values of TopDom parameter k. Non-border SNPs are defined as the complementary set of SNPs, yielding the network labelled ‘non-border’. The networks labelled ‘border intergenic’, and ‘non-border intergenic’ are obtained when the additional condition of being intergenic is imposed on the shared SNPs. Note that these graphs are not disjoint; two diseases can be linked in two or more of these diseasome networks.

The level of clustering (density of internal links) of various subgraphs, compared to a situation of random wiring, has been estimated using the notion of network coherence, defined as the fraction of connected nodes within the subgraph, z-transformed with a null model of randomly drawn node sets of the same size (see *Methods*).

The considered subgraphs correspond to sets of diseases: cancers, non-cancer diseases, cancers whose associated SNPs are preferentially located in TAD borders for a majority of values of k (‘enriched cancer’) or not (‘not enriched cancer’) and similar definitions for enriched and not enriched non-cancer diseases. Network coherence provides an absolute quantification, here computed for the 6 subgraphs in the four diseasome networks. A random subgraph would have a vanishing network coherence, a positive value indicates a high internal connectivity while a negative value reveals that the subgraph is less connected than a random set of nodes in the overall network.

Distance threshold	10 kb	20 kb	200 kb	1 Mb	Total number of pairs of daSNPs
Cancers, any daSNPs	8	11	32	66	9909
Cancers, no enrichment, border daSNPs	0	0	0	0	47
Cancers, with enrichment, border SNPs	2	4	6	9	1196
Non-cancer diseases, any daSNPs	18	28	94	149	14353
Non-cancer diseases, no enrichment, border daSNPs	0	1	2	3	212
Non-cancer diseases, with enrichment, border daSNPs	19	24	44	59	1147

Supplementary Table 2 : **Analysis of pairwise distances between daSNPs**.
For each disease, we computed the number of pairs of associated SNPs located at a distance closer than a threshold, equal to either 10kb (size of a bin), 20kb (size of a TAD border), 200kb or 1Mb (upper bounds on LD range). An average was then taken over several categories of diseases (cancers and non-cancer diseases, displaying or not TAD border enrichment) distinguishing when pairs are between any SNPs or only between border SNPs (the borders being determined as previously in the typical case of IMR90, data from [S2], TopDom parameter k=10). These numbers(rounded to the lower integer) show that the potential contribution of pairs of SNPs in strong LD is negligible in the interpretation of our enrichment results.

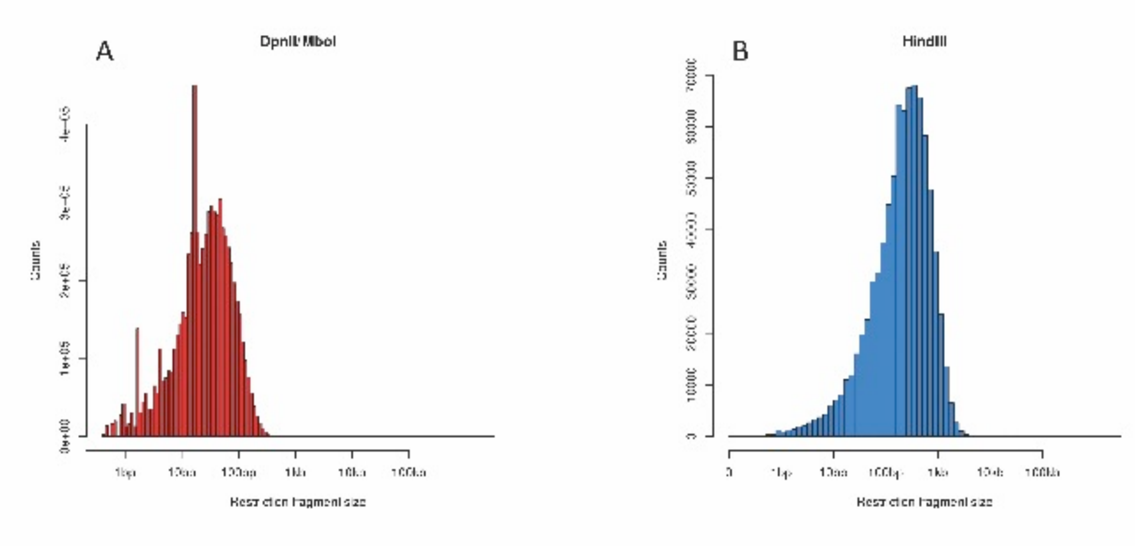

Supplementary Fig 8. Restriction fragment size distributions.
The distribution of the fragment size (size in log scale on the horizontal axis) has been determined from the positions of the restriction sites on the genome (using *digest_genome* script from HiC-Pro toolbox [S8]) on the NCBI genome version) for: (A) the restriction enzymes MboI or DpnII, producing the same fragments (the enzymes recognize the same sequence) and (B) the restriction enzyme HindIII producing larger fragments (note the different scale for the counts on the vertical axis). These distributions show that all restriction fragments obtained with MboI or DpnII have a size below 1kb, whereas a non-negligible percentage of DNA fragments obtained with HindIII have a size larger than 1kb, however below 10kb, indicating that Hi-C resolution is mostly limited by sequencing depth.

Supplementary File 2: Typical numbers of diseases and SNPs involved in the analysis

About 350 Mb are located within TAD borders, representing about 12% of the genome and 13% of the number of base pairs located in TADs. However, this number of base pairs located in TAD borders varies with the value of the parameter *k* used in the TAD caller TopDom, roughly decreasing when *k* increases. Exact values in the case of IMR90 cell type, data from [S2], are given in Supplementary Table 3.

Window size *k*	TAD border contents relative to genome	TAD border contents relative to TADs
3	12.8%	14.4%
4	14.1%	15.8%
5	14.3%	16%
6	14.1%	15.7%
7	13.6%	15.2%
8	12.9%	14.4%
9	12.3%	13.7%
10	11.7%	13.1%
11	11%	12.3%
12	10.5%	11.8%
13	10.1%	11.3%
14	9.7%	10.8%
15	9,2%	10.3%
16	8.9%	10%
17	8.6%	9.6%
18	8.3%	9.3%
19	8.1%	9%
20	7.8%	8.7%

Supplementary Table 3: **Genome fraction located in TAD borders.**
The fraction of the genome (resp. of the total number of base pairs in TADs) located in TAD borders is given for different values of the parameter *k* (window size) of the TAD caller TopDom (underlying Hi-C data from [S2], IMR90 cell type).

Only the statistical analysis could support statements about TAD border enrichment in daSNPs. However, to give a feeling of the orders of magnitude, we give below some typical numbers (by default, TAD borders are those determined with k=10 for the IMR90 cell type, data from [S2]).

449 EFOs have been considered in the study, among which 71 cancers (that is, 16%). . The overall number of base pairs located in TAD borders and border SNP counts vary with the datasets, as described in Supplementary Table 4. The number of disease-associated SNPs (without multiplicity) is 21,183 among which about 2800 (13%) are located in TAD borders, resp. 3,319 cancer-associated SNPs among which about 470 (14%) are located in TAD borders.

There is on average 9 border SNPs (rounded value) per disease, with no significant difference between cancers and non-cancer diseases. The standard deviation of the number of border SNPs per disease is larger than its mean (here 23 compared to 9) which reflects a broad distribution and the presence of outlier extreme values (here 227)

The mean number of border SNPs associated with a disease dramatically increases when considering only diseases displaying TAD border enrichment in their associated SNPs, reaching on average 30 border SNPs for cancers and a similar value (29) for non-cancer diseases, while the mean decreases to 5 (resp. 7) for cancers (respect. non-cancer diseases) that do not display TAD border enrichment. We again underline that these figures are not sufficient to assess TAD border enrichment, and they can only motivate further statistical tests.

8,438 (40%) disease-associated SNPs are intergenic. 1,058 are intergenic and located in TAD borders. 1,275 are intergenic and associated with cancer. 176 are intergenic, associated with cancer and located in TAD borders.

11,552 (55%) disease-associated SNPs are intronic. 1,508 are intronic and located in TAD borders. 1,854 are intronic and associated with cancer. 257 are intronic, associated with cancer and located in TAD borders.

1,171 (5%) disease-associated SNPs are exonic. 223 are exonic and located in TAD borders. 185 are exonic and associated with cancer. 35 are exonic, associated with cancer and located in TAD borders

Data set	TAD border contents (w.r.t. entire genome)	TAD border contents (w.r.t. all TADs)	border SNP count	fraction of border SNPs (w.r.t. all daSNPs)
Dixon et al 2012<br>H1 hESC<br>HindIII enzyme	8.7%	9.7%	1969	9.3%
Dixon et al 2012<br>IMR90<br>HindIII enzyme	8.8%	9.9%	2018	9.5%
Jin et al 2013<br>IMR90<br>HindIII enzyme	7.7%	8.6%	1834	8.7%
Selvaraj 2013<br>GM12878<br>HindIII enzyme	12.2%	13.7%	2552	12%
Dixon et al 2015<br>H1 hESC,<br>HindIII enzyme	13.1%	14.7%	3179	15%
Dixon et al 2015<br>H1_ME,<br>HindIII enzyme	13.6%	15.3%	3223	15%
Dixon et al 2015<br>H1_NP<br>HindIII enzyme	14.4%	16.1%	3398	16%
Dixon et al 2015<br>H1_TB<br>HindIII enzyme	12.8%	14.3%	3087	15%
Dixon et al 2015<br>H1_MS<br>HindIII enzyme	12.6%	14%	3053	14%
Rao et al 2014<br>GM12878<br>DpnII enzyme	11.7%	13.1%	3056	14%
Rao et al 2014<br>GM12878<br>MboI enzyme	10.8%	12%	2789	13%
Rao et al 2014<br>IMR90<br>MboI enzyme	11.7%	13.1%	3034	14%
Rao et al 2014<br>HMEC<br>MboI enzyme	13.1%	14.7%	3408	16%
Rao et al 2014<br>NHEK<br>MboI enzyme	15%	16.8%	3698	17%
Rao et al 2014<br>HUVEC<br>MboI enzyme	11.8%	13.2%	3005	14%

Supplementary Table 4: Genome fraction and number of SNPs in TAD borders for different datasets. The fraction of the genome (resp. of the total number of base pairs in TADs) and the number (resp. the fraction) of disease-associated SNPs located in TAD borders is given for different data sources (for a value *k*=10 of the TopDom window-size parameter *k*). Unless otherwise stated, data from [S2] were obtained from experiments using MboI enzyme, whereas all other datasets were obtained from experiments using HindIII enzyme.

References in the Supplementary Material

S1 Shin H, Shi Y, Dai C, Tjong H, Gong K, Alber F, et al. TopDom: an efficient and deterministic method for identifying topological domains in genomes. Nucleic Acids Res. 2016;44: 1-13.

S2 Rao SS, Huntley MH, Durand NC, Stamenova EK, Bochkov ID, Robinson JT. A 3D map of the human genome at kilobase resolution reveals principles of chromatin looping. Cell. 2014;159: 1665–1680.

S3 Benjamini Y, Hochberg Y. Controlling the false discovery rate: A practical and powerful approach to multiple testing. J Roy Stat Soc B Met. 1995;57: 289-300

S4 Dixon JR, Selvaraj S, Yue F, Kim A, Li Y, Shen Y, et al. Topological domains in mammalian genomes identified by analysis of chromatin interactions. Nature .2012;485: 376-380

S5 Jin F, Li Y, Dixon JR, Selvaraj S, Ye Z, Lee AY., et al. A high-resolution map of the three-dimensional chromatin interactome in human cells. Nature. 2013;503: 290.

S6 Selvaraj S, Dixon JR, Bansal V, Ren B. Whole-genome haplotype reconstruction using proximity-ligation and shotgun sequencing. Nat Biotechnol. 2013;31: 1111-1118

S7 Dixon JR, Jung I, Selvaraj S, Shen Y, Antosiewicz-Bourget JE, et al. Chromatin architecture reorganization during stem-cell differentiation. Nature. 2015;518: 331-336.

S8 Servant N, Varoquaux N, Lajoie BR, Viara E, Chen CJ, Vert JP, et al. HiC-Pro: an optimized and flexible pipeline for Hi-C data processing. Genome Biol. 2015;16: 259.
